# Selection of prescription isodose line for brain metastases treated with volumetric modulated arc radiotherapy

**DOI:** 10.1002/acm2.12761

**Published:** 2019-12-03

**Authors:** Yuan Xu, Pan Ma, Yingjie Xu, Jianrong Dai

**Affiliations:** ^1^ Department of Radiation Oncology National Cancer Center/National Clinical Research Center for Cancer/Cancer Hospital Chinese Academy of Medical Sciences and Peking Union Medical College Beijing China

**Keywords:** brain metastases, gradient index, prescription isodose line, VMAT

## Abstract

**Purpose:**

To exploit the optimal prescription isodose line (IDL) for brain metastases treated with volumetric modulated arc radiotherapy (VMAT) as there is no consensus on the selection of IDL with VMAT.

**Methods and materials:**

Eighteen patients with 20 brain tumors, who were treated with VMAT, were enrolled in this study. For each tumor of every patient, five plans were designed with IDL ranging from 50% to 90% in 10% increments. Different IDLs were obtained through adjusting the constraint parameters during planning optimization. Prescription dose (10 × 5 Gy) were identical for all plans, and the plans were compared in terms of gradient index (GI), conformity Index (CI), V26 Gy/V_PTV_, and V32 Gy/V_PTV_ in normal brain tissue, which correlate to radiation necrosis.

**Results:**

IDL with lowest GI has a median value of 60.0% (ranging from 50% to 80%). Except for one tumor with volume larger than 10 cc, the IDL with lowest GI varies from 50% to 70%, which depends on the shape of PTV, location, and whether the target volume is adjacent to crucial OAR. Moreover, there is no significant difference for CI with varying IDL plans. The average V26 Gy/V_PTV_ and V32 Gy/V_PTV_ in normal brain tissue 60% IDL plans are 27.3%, 31.7% lower than 90% IDL plans separately (*P* < 0.05). However, by further decreasing IDL from 60% to 50%, the average V26 Gy/V_PTV_ and V32 Gy/V_PTV_ may increase comparing with 60% IDL plans (*P* > 0.05). Furthermore, a lower IDL is found to result in higher mean dose to the target volume (*P* < 0.05).

**Conclusions:**

Plans using VMAT with PTV smaller than 10 cc tend to be optimal with IDL around 60–70% for lower GI, smaller V26 Gy/V_PTV_, V32 Gy/V_PTV_ in normal brain tissue, and higher mean dose in tumor comparing with high IDL plans which have potential benefit in reducing risk of radiation necrosis and increasing the local control. However, IDL lower than 60% is not recommended for the disadvantage of increasing V26 Gy/V_PTV_ and V32 Gy/V_PTV_ in normal brain tissue.

## 1 INTRODUCTION

Statistically, brain metastases are found in 20–40% patients with cancer.^1^ Radiotherapies such as whole brain radiation therapy (WBRT) and stereotactic radiosurgery (SRS) are routine treatments for patients with brain metastases. However, comparing with SRS, WBRT has toxicity concerns and no benefit of survival.^2^, ^3^ Therefore, SRS seems to be more popular in the radiotherapy of brain metastases. Unfortunately, for larger or irregular targets or targets abutting critical organ at risk (OAR) which can bring unacceptable toxicity, SRS is not suitable anymore.^4^, ^5^ According to radiation biology principle, fractionated stereotactic radiotherapy (SRT) makes it possible to better protect the normal tissue with similar local control comparing with single fraction.^6^, ^7^ In practice, fractionated SRT combines the advantage of steep dose gradients in SRS and lower toxicity in normal tissue by fractionating.

Historically, intracranial SRS is treated mainly by Gamma Knife (GK). As technology advances, robotic radiosurgery CyberKnife (CK) and conventional linac are also developed in SRS treatment.^8^ For linac‐based SRS/SRT, non‐coplanar 3D conformal radiotherapy or dynamic conformal arcs technology are mostly utilized for treatment.^9^ Volumetric modulated arc radiotherapy is an innovative technique developed in recent years. By rotating the gantry with moving multileaf collimator (MLC), high‐dose conformity and treatment efficiency can be achieved by linac‐based VMAT.^10^ Due to its superior performance, VMAT is introduced to SRS, SRT, and also stereotactic body radiation therapy (SBRT).^11^ For fractionated SRT, it is reported in a planning study that VMAT plan could outperform GK plan with better conformity and lower doses to the OARs for large or irregular targets.^12^

A fundamental principle in radiotherapy is to achieve effective dose delivering in tumor while minimizing the toxicity to normal tissue. It is somehow more crucial in SRS/SRT as a high dose per fraction is delivered which requires high precision and better protection of normal tissue.^13^ Consequently, a homogeneous dose distribution in planning target volume (PTV) is not necessary for SRS/SRT, and a steep dose falloff and a high conformal dose distribution are important. As inhomogeneity is not regarded as a problem in SRT like other conventional VMAT plans, the selection of prescription isodose line will have impact on the dose distribution and normal tissue sparing. It is recommended in ICRU report 91 which is specified for stereotactic treatments that absorbed dose is prescribed to the isodose surface enclosing optimal percentage volume of PTV while limiting the dose to planning OAR volume.^14^ However, it is not defined how to choose the optimal IDL, and there is also no consensus among institutes in clinical application.

In general, IDL around 50% is often used in GK plans.^15^ Nevertheless, the selection of IDL for CK is more flexible ranging from 5090%. A higher IDL of 80–90% are normally used in linac‐based SRS, but IDL ranging from 50–90% has been utilized in clinical practice. Recently, there are several studies concerning the selection of IDL in SRS. It is reported by Bo zhao et al. that 50–75% IDL may be optimal for linac‐based SRS using non‐coplanar dynamic conformal arcs by mainly referencing GI.^16^ For CK‐based SRS, Qianyi Xu et al. concluded that lower IDL plans (49.6 ± 2.1%) outperform high IDL plans (88.6 ± 1.3%) with lower dose of normal brain tissue and better CI.^17^ A new index named dose‐dropping speed was proposed by Q. Zhang et al. to evaluate the impact of IDL in linac‐based SRS using non‐coplanar dynamic conformal arcs as well.^18^ It is found that the dose‐dropping speed increases with decreasing IDL and reach a plateau around 60–70% IDL. Nevertheless, there is no study reported concerning the selection of IDL in linac‐based intracranial VMAT technique.

In this study, VMAT plans with IDL ranging from 50% to 90% were optimized for each tumor. For 20 brain metastases, 100 plans in total were generated to explore the optimal IDL. Several metrics including GI, CI, V26 Gy, and V32 Gy in normal brain which may induce radiation necrosis in normal brain were compared.

## 2 MATERIALS AND METHODS

### 2.1 Patients and planning technique

Under the approval of the institutional review board, 18 patients with 20 brain metastases were retrospectively studied in our research. The volume of PTV ranges from 0.41 cc to 11.71 cc (mean 3.76 ± 2.81 cc). For comparison, the prescription dose of these patients is identical with 50 Gy delivering in ten fractions. The plans were performed in Pinnacle treatment planning system version 9.1 (Philipps Healthcare, Best, Netherlands). The planning CT data were scanned by a Somatom Definition AS 40 (Siemens Healthcare, Forchheim, Germany) or Brilliance CT Big Bore (Philips Healthcare, Best, Netherlands) with 2 mm thickness. All plans were created on an Edge radiosurgery system (Varian Medical System, Palo Alto, CA, USA) which is a linear accelerator system specially designed for high accuracy radiosurgery with extra‐fine 2.5 mm MLC leaves for beam shaping.

For each tumor, two arcs using VMAT technique were used for planning. As recommended in RTOG 0320/9508 for SRS, 50–90% IDL should encompass the margin of the metastases,^19^, ^20^ and IDLs lower than 50% is not commonly used in clinical practice in linac‐based VMAT. Therefore, five plans with varying IDL of 50%, 60%, 70%, 80%, and 90% were optimized for each tumor. This is realized by constraining the volume of 50% prescription dose (which means “Max DVH” of 25 Gy in Pinnacle system) in normal tissue encompassing the PTV in the inverse optimizer and keeping other parameters the same. During planning, flattening filter free (FFF) mode was utilized with maximum dose rate of 1400 MU/min, and 0.2 mm fine grid was used for dose calculation. For comparing, all plans were normalized to the same prescription dose and guarantee identical 98% coverage of PTV by prescription dose.

### 2.2 Evaluation parameters

Several parameters were used in our study to evaluate the optimal plans for VMA treatment. As recommended in the ICRU report 91,^14^ the dose gradient index is used to characterize the dose falloff in radiosurgery, which is defined as:

(1) $$\text{GI}=\frac{{\text{PIV}}_{\text{half}}}{\text{PIV}}$$

where ${\text{PIV}}_{\text{half}}$ represents the prescription isodose volume enclosing half the prescription dose, and PIV represents the prescription isodose volume. Apparently, a lower GI value shows steep dose falloff in normal tissue. Conformity index is the ratio of the prescription dose volume to the PTV volume. It is used to characterize the degree to which the prescription dose conforms the PTV.^21^

In intracranial SRT/SRS, radiation necrosis should be considered which is associated with V10 Gy and V12 Gy in normal brain tissue if the dose is delivered in a single fraction.^22^ However, for fractionated treatment, the equivalent biological dose should be calculated by using the concept of biological effective dose (BED). BED of a n fractions schedule with dose of d per fraction can be calculated as:^23^

(2) $$\text{BED}=n\cdot d(1+\frac{d}{\alpha /\beta })$$

where the value of α/β is dependent on the tissue and is assuming 2 Gy for normal brain tissue. For example, for a single fraction, the BED of 10 Gy in brain tissue is 60 Gy. By assuming a same BED received, the total dose with n fractions can be calculated as:^24^

(3) $$D=n(-\frac{\alpha /\beta }{2}+\sqrt{{\left(\frac{\alpha /\beta }{2}\right)}^{2}+\frac{\alpha /\beta \cdot \text{BED}}{n}})$$

Therefore, for a radiotherapy course of 10 × 5 Gy, the equivalent biological dose should be 26 Gy, 32 Gy corresponding for 10 Gy, 12 Gy delivering in a single fraction separately. In our study, V26 Gy and V32 Gy are compared to evaluate the dependence of normal tissue sparing on the IDL. Statistical analysis was performed by Wilcoxon signed‐rank test to characterize the significance of related parameters’ variations with different IDL plans comparing with referencing 90% IDL plans.

## 3 RESULTS

**Figure 1:**
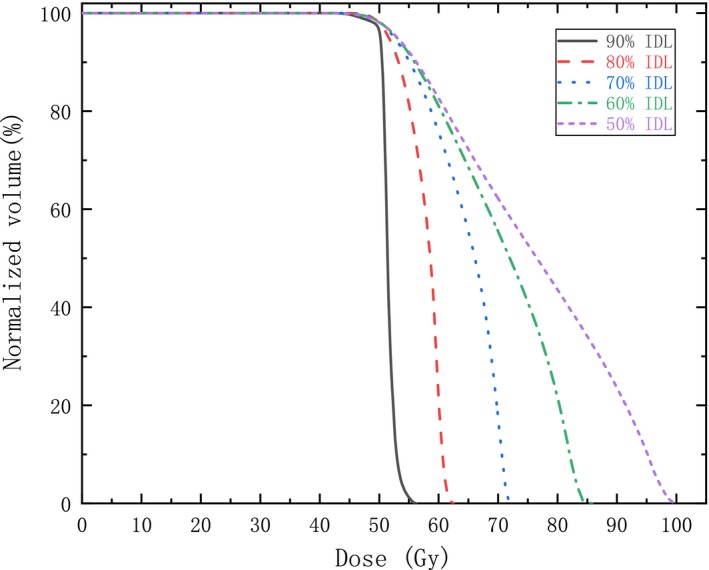
The dose volume histogram of PTV with varying IDL. IDL, isodose line; PTV, planning target volume.

**Figure 2:**
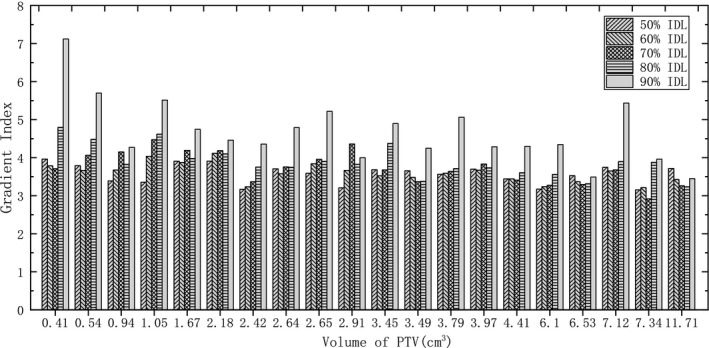
The gradient index for individual lesions sorted to size.

**Figure 3:**
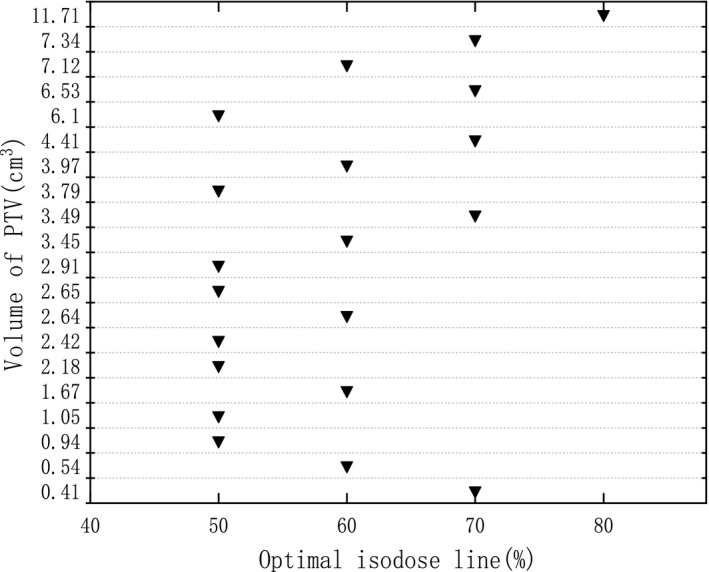
Isodose line with lowest GI for individual tumors sorted to size.

For each case, five plans with IDL varying from 50% to 90% were optimized by changing the constraint parameters in the inverse optimizer using VMAT technique. Afterwards, all plans were normalized to have the identical prescription dose (10 × 5 Gy) covering 98% of the PTV. It is an example shown in Fig. 1 that dose distribution in PTV is significantly different with varying IDL. It is apparent that the maximum dose increases with lowering the IDL. In Fig. 2, GI calculated with Eq. (1) is illustrated for each tumor with varying IDL. As a whole, there is a trend that GI decreases with IDL declining from 90% initially, and increases afterwards with further decreasing the IDL if GI reaches the minimum. Moreover, it can also be seen in some cases, GI declines all the way with IDL decreasing from 90% to 50% because the minimum GI may appear at an IDL lower than 50% which is not considered in this study and not commonly used in VMAT planning. The IDL with the lowest GI is shown in Fig. 3 for all tumors. The median IDL for lowest GI is 60.0% (range from 50% to 80%). Except for one tumor with volume greater than 10 cc, the lowest GI is varying from 50% to 70% in all other cases. It seems that smaller tumors have the lowest GI occurs at a lower IDL. Nevertheless, it depends on the shape of PTV, location, and whether the target volume is adjacent to crucial OAR. Statistical analysis shows that GI with IDL of 50%, 60%, and 70% have a significant difference comparing with 90% IDL plans (*P* < 0.05). The CI for all plans is clinically acceptable, but have no significant differences with varying IDL.

**Figure 4:**
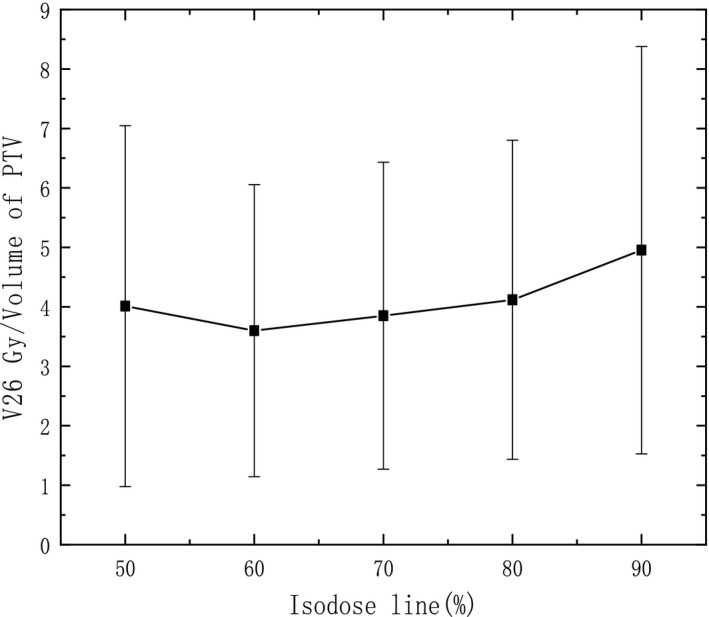
V26 Gy/V_PTV_ varying with IDL.

**Figure 5:**
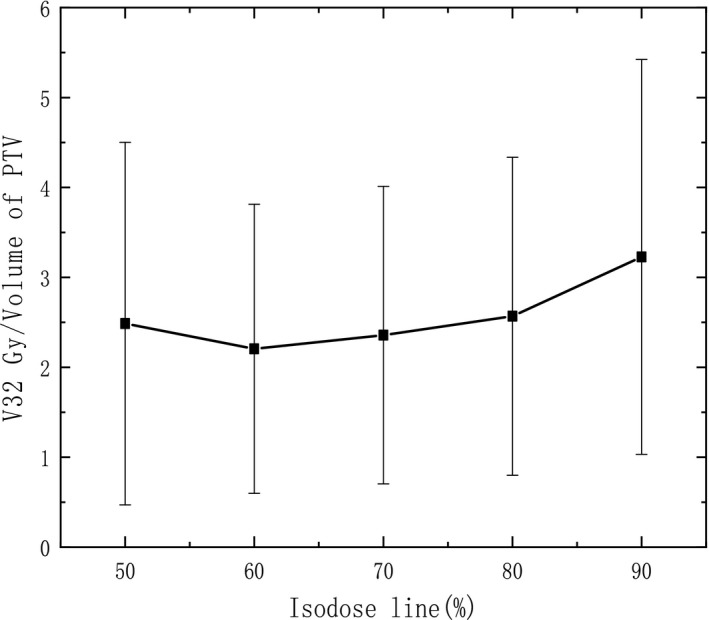
V32 Gy/V_PTV_ varying with IDL.

Actually, the investigation of V26 Gy/V_PTV_, V32 Gy/V_PTV_ (V_PTV_ means the volume of PTV) in normal brain is also carried out to show normal tissue sparing. It is illustrated in Fig. 4 and Fig. 5, the mean V26 Gy/V_PTV_, V32 Gy/V_PTV_ decrease obviously with IDL varying from 90% to 70%, and reach a plateau around 70–60%. Comparing with 90% IDL plans, the V26 Gy/V_PTV_, V32 Gy/V_PTV_ are 27.3%, 31.7% lower with 60% IDL plans, separately (*P* < 0.05). However, if the IDL is lower than 60%, the V26 Gy/V_PTV_, V32 Gy/V_PTV_ in normal brain seems to slightly increase due to higher MU is needed to achieve a higher dose delivering inside the PTV (*P* > 0.05). This result is agreed with the investigation of GI that optimal IDL around 60% can provide better normal tissue sparing comparing with high IDL plans.

**Figure 6:**
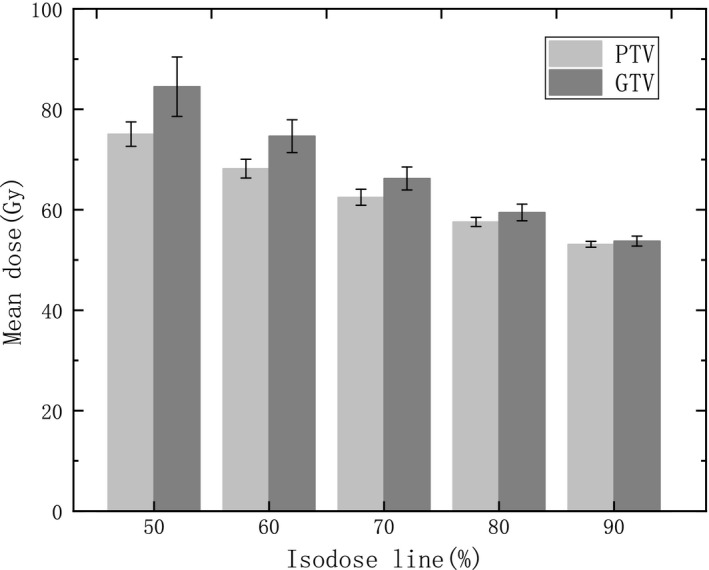
The mean dose of PTV and GTV varying with IDL. IDL, isodose line; PTV, planning target volume.

Except for the dose delivering in normal tissue, the dose distribution in target volume should also be evaluated as it is associated with local control of tumor. As can be seen in Fig. 6, the mean dose of PTV and GTV increases obviously from 53.1 ± 0.6 Gy, 53.8 ± 1.0 Gy to 75.1 ± 2.4 Gy, 84.5 ± 5.9 Gy separately with IDL decreasing from 90% to 50%. This means that more dose is delivered to the tumor with lower IDL. The mean D98% in PTV is similar for all IDL plans, and D2% and homogeneity Index increases with IDL declining which is reasonable according to the definition of IDL.

## 4 DISCUSSION

**Figure 7:**
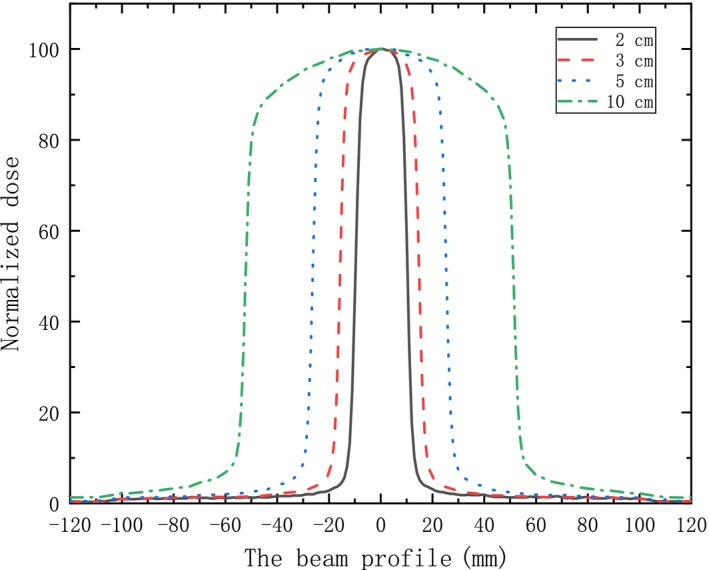
The measured FFF beam profiles of MLC defined square fields with varying sizes of 2 × 2, 3 × 3, 5 × 5, 10 × 10 cm. (Beam model with source axis distance 100 mm at 5 cm depth). FFF, flattening filter free; MLC, multileaf collimator.

The optimization process of a VMAT plan is different from that of 3D conformal technique. In conformal plans, varying IDL plans can be achieved by forward planning by adjusting the MLC margin with suitable filed arrangement. However, it is more complicated for VMAT planning. There is no uniform margin for each field, and the beam distribution is optimized by sliding MLC and varying intensity with rotating gantry, which is realized by inverse optimization algorithm in treatment planning system by setting different objectives for the optimizer. Nevertheless, relationship still can be found between the margin and IDL in VMAT plan. It can be seen in the planning system that margins for some control points tend to be smaller with decreasing IDL. This can be somehow explained by profiles illustrated in Fig. 7, and a steeper dose drop‐off can be formed with smaller field. For a low IDL plan, the margin seems to be smaller to reduce the 50% prescription dose volume in the normal tissue. It is also found that when margins for all fields is approaching zero, the GI almost reaches lowest value. Afterwards, plans with further lower IDL should be realized with increasing total MU to get a higher dose in the PTV, which may also lead to the increasing of dose in normal tissue. Bo Zhao et al. reported that the optimal GI increased obviously with smaller target volume in linac‐based dynamic conformal arc planning.^16^ A similar trend is also found in VMAT planning, but the impact is not so apparent comparing with conformal planning as VMAT has more optimization parameters to shape the beam distribution to achieve specific IDL.

**Figure 8:**
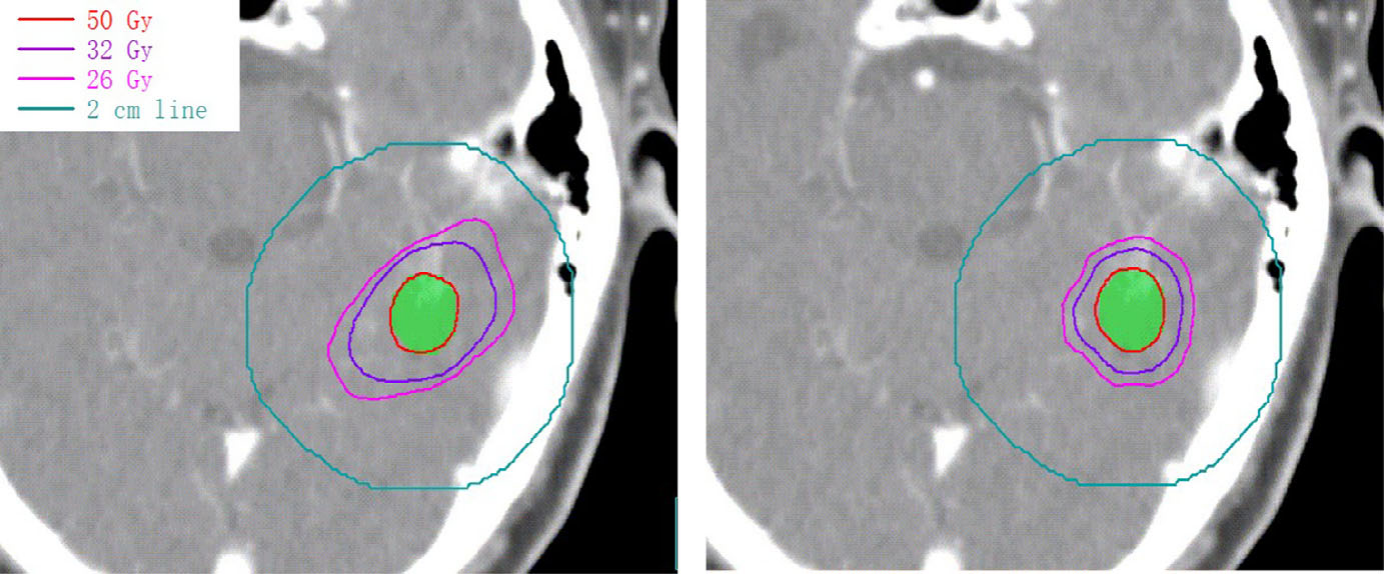
Comparison of the dose distribution for a representative patient with 90% IDL plan (left) and 60% IDL plan (right). IDL, isodose line.

Radiation necrosis should also be considered in intracranial radiation therapy. It is reported that the risk of radiation necrosis has relationship with V8 Gy‐16 Gy in normal brain tissue.^22^ Furthermore, it is illustrated by Blonigen et al. that the risk of radiation necrosis increases significantly with a threshold volume of a midpoint volume of 10.5 cc for V10 Gy and 7.9 cc for V12 Gy. In our study, the corresponding dose with the equivalent biological dose of 10 Gy and 12 Gy delivering in one fraction was calculated to be 26 Gy and 32 Gy for a course of 10 × 5 Gy separately for comparison in fractionated SRT. It is shown in Fig. 4 and Fig. 5 that the mean V26 Gy, V32 Gy are 13.78 ± 7.97 cc, 8.87 ± 4.68 cc separately with 90% IDL plans, which both exceed the threshold volume indicated by Blonigen et al. Fortunately, 60%, 70% IDL plans outperform 90% IDL plans in both V26 Gy and V32 Gy, and they are lower or close to the threshold volume. This means that the risk of radiation necrosis can be reduced obviously with 60–70% IDL comparing with 90% IDL. It is more apparent in an example shown in Fig. 8, the volume encompassed by V26 Gy and V32 Gy is much smaller comparing 60% IDL plan (right) with 90% IDL plan (left). But it also can be seen that if the IDL is lower than 60%, the V26 Gy and V32 Gy may increase slightly due to higher MU is delivered for low IDL plans. Moreover, a too low IDL plan should be avoided for safety season as extremely high dose point (e.g., 100 Gy for 50% IDL) occurs, and uncertainty of setup or beam delivering may cause unexpected problem. A higher dose in adjacent OAR is also a concern associating with lower IDL plans. Generally, lower IDL is associated with higher MU, which is 1786 ± 370 MU for 50% IDL plans, and 850 ± 163.5 MU for 90% IDL plans. Therefore, the treatment efficiency may decline if too low IDL is used for clinic.

Another potential advantage of low IDL plans is that local control of brain metastases may be improved with increasing dose delivering in the target volume.^25^ It is reported by Rades et al. that the tumor control possibility increases obviously with delivering boost dose in target volume.^26^ Although more clinical study is needed, potential benefits exist for improving the tumor control by increasing the dose delivering to the target volume. It is shown in our study that the mean dose of PTV and GTV increases obviously with IDL decreasing. According to Linear Quadratic model, the mean BED in PTV and GTV increases from 81.3 Gy, 82.7 Gy to 131.5 Gy, 155.9 Gy separately.^27^ As inhomogeneity is somehow accepted in SRT planning, a higher dose in PTV and GTV is possible with lower IDL plan to potentially improve the local control of tumor. Moreover, as higher dose delivering can be realized by utilizing low IDL, a prescription dose de‐escalation may be possible with low IDL plan.^28^

The results of the optimal IDL for VMAT plans in our study are consistent with the conclusion of the study concerning linac‐based dynamic conformal arc plans, which also indicates that lower IDL plans have lower GI and steeper dose falloff comparing with high IDL plans.^16^, ^18^ Moreover, it is further proposed in our study that a too low IDL (<60%) should be avoided as it may increase the risk of radiation necrosis and also for safety concern.

## 5 CONCLUSIONS

The impact of IDL selection for VMAT plans was studied. For lesions smaller than 10 cc, plans with IDL around 60–70% tend to be optimal, which outperforms 90% IDL plans with lower GI and lower risk of radiation necrosis in normal brain tissue. Better local control with higher dose in tumor is also a potential benefit associate with lower IDL. Nevertheless, too low IDL plan is not recommended considering treatment efficiency, higher risk of radiation necrosis, and also setup uncertainty problem with very high dose delivering.

## CONFLICT OF INTEREST

There is no conflict of interest to declare.
